# Integrated Seroprevalence and Genome‐Based Study of SARS‐CoV‐2 Viral Strains in N′Djamena: Insights Into Chad's COVID‐19 Epicenter

**DOI:** 10.1002/jmv.70234

**Published:** 2025-02-18

**Authors:** Mathiew Hota, Andrillene L. D. Wondeu, Mahamat F. Abakar, Koutaya Dezoumbe, Fatima Abdelrazakh, Sabrina Atturo, Nathan Naïbeï, Giulia Cappelli, Franck Mennechet, Fissou H. Yandai, Djamal H. Abdallah, Zongo R. F. Edgard, Abdoulaye Boukar, Choroma A. Moussa, Issa M. Yaya, Mahamat I. Hamad, Nontegyol Armand, Netalar Honorine, Kayanlengar Frederic, Adam A. Moustapha, Yanda M. Daniel, Adam M. Alim, Mahamat Grene, Oumaima Djarma, Noubaramadji Y. Suitombaye, Amine Akouya, Ouchemi Choua, Guy R. T. Dzomo, Djallaye Djimtoïbaye, Vittorio Colizzi, Mahamat A. Moussa, Marta Giovanetti

**Affiliations:** ^1^ Department of Laboratories Ministry of Public Health N'Djamena Chad; ^2^ LAGET Major Tropical Epidemics Laboratory Bon Samaritain University Hospital N'Djamena Chad; ^3^ Evangelical University of Cameroon Bandjoun Cameroon; ^4^ Institut de Recherche en Elevage pour le Développement (IRED) N'Djamena Chad; ^5^ MAGIS Foundation N'Djamena Chad; ^6^ Community of Friends of Computing for Development “CAID‐Tchad N'Djamena Chad; ^7^ Institute for Biological Systems (ISB)‐CNR Monterotondo Italy; ^8^ University of Montpellier, Pathogenesis and Control of Chronic an Emerging Infections (PCCEI), INSERM U1058, French Blood Establishment (EFS) Montpellier France; ^9^ OMS Tchad, Assistant Laboratoire COVID ‐19 N'Djamena Chad; ^10^ King Faycal Hospital N'Djamena N'Djamena Chad; ^11^ Abeché University Hospital Laboratory Abeché Chad; ^12^ PMTCT/HIV Bol Health District N'Djamena Chad; ^13^ Biltine Provincial Hospital Biltine Chad; ^14^ Doba Provincial Hospital Doba Chad; ^15^ Moundou Provincial Hospital Moundou Chad; ^16^ Sarh Provincial Hospital Sarh Chad; ^17^ Ati Provincial Hospital Ati Chad; ^18^ Bongor Provincial Hospital Bongor Chad; ^19^ Pala Provincial Hospital Pala Chad; ^20^ Mongo Provincial Hospital Mongo Chad; ^21^ Department of Infectious Diseases/National University Hospital of Reference Université Bon Samaritain N'Djamena Chad; ^22^ Faculty of Human Health Sciences, N'djamena University N'djamena Chad; ^23^ National University Hospital of Reference N'Djamena Chad; ^24^ Le Bon Samaritain University Hospital Complex N'Djamena Chad; ^25^ Chantal Biya International Reference Center for Research on HIV/AIDS Prevention and Management (CIRCB) Yaoundé Cameroon; ^26^ University of Rome “Tor Vergata” Rome Italy; ^27^ Department of Sciences and Technologies for Sustainable Development and One Health Università Campus Bio‐Medico di Roma Roma Italy; ^28^ Instituto Rene Rachou, Fundação Oswaldo Cruz, Minas Gerais Belo Horizonte Brazil; ^29^ Climate Amplified Diseases And Epidemics (CLIMADE) Belo Horizonte Brazil

**Keywords:** Chad, genomic surveillance, SARS‐CoV‐2, seroprevalence

## Abstract

The COVID‐19 epidemic has shown regional variations in transmission and outcomes. As a primary hotspot in Chad, N'Djamena is crucial for comprehensive epidemiological investigation. Our study employed two methodologies: seroprevalence data collection and whole‐genome sequencing of SARS‐CoV‐2 strains. This dual approach assessed population exposure and virus genetic diversity. Seroprevalence data indicated broader exposure than confirmed cases suggested, and genome sequencing identified multiple strains, including globally recognized variants of concern. Integrating these data provided insights into transmission dynamics, potential herd immunity thresholds, and the impact of specific variants on disease progression. Our findings underscore the importance of integrated, multidisciplinary research in infectious disease epidemiology and inform targeted public health strategies, including social measures and vaccination, to combat infectious diseases in N'Djamena.

## 1 Introduction

Since the onset of the COVID‐19 pandemic, the severe acute respiratory syndrome coronavirus 2 (SARS‐CoV‐2) has undergone numerous mutations, resulting in genetic variations that were not initially anticipated. Different strains of the virus have emerged globally, and some have raised concerns regarding their potential impact on infectivity, immune evasion, and reinfection risk [1]. Viral sequencing analyses suggest that a significant number of the SARS‐CoV‐2 strains observed in Europe and America have similarly been identified in Africa. This alignment underscores the potential critical influence of human mobility in facilitating the spread and introduction of these viral strains into previously unaffected regions [2]. The patterns of mortality and morbidity linked to the virus vary significantly between continents. In addition, the distribution of these rates varies considerably within each continent. Recent research has illustrated the influence of specific SARS‐CoV‐2 variants, termed as variants of concern (VOCs), on altering clinical outcomes [3]. These VOCs play a crucial role in augmenting the virus's transmissibility, and they have been linked to heightened morbidity and mortality from COVID‐19, providing a deeper understanding of the multifaceted nature of the pandemic [3]. In Africa, marked variations in COVID‐19 incidence have been recorded across southern, northern, central, and other regions [4, 5]. Within sub‐Saharan Africa, available data on circulating viral strains is limited. However, seroprevalence studies have demonstrated that SARS‐CoV‐2 has been widely present, despite the relatively low morbidity and mortality in many countries [6]. When considering Africa's geographical distribution, meta‐analysis results suggest that Central and Western Africa exhibit higher seroprevalence rates (26.5% and 22.0%, respectively) compared to Southern (20.7%), Eastern (12.1%), and Northern (6.5%) regions [7]. In Chad, between January 2020 and September 2023, 7698 confirmed COVID‐19 cases and 194 deaths were reported according to the World Health Organization [8]. N'Djamena, the capital city of Chad, was the most severely affected area during the COVID‐19 pandemic. The city's population increased from 1 476 000 in 2021 to 1 533 000 in 2022, during which varying rates of morbidity and mortality were recorded. N'Djamena [9, 10] experienced significant epidemiological fluctuations in its COVID‐19 indicators between 2021 and 2022. In 2021, epidemiological data showed 4070 confirmed cases, resulting in a morbidity rate of 0.275%. During that period, the city also recorded 80 fatal virus‐related complications, corresponding to a mortality rate of 1.965%. By contrast, in 2022, there was a significant decline in the number of confirmed cases, with only 1470 cases reported, leading to a reduced morbidity rate of 0.095%. The mortality rate also dropped substantially, with only 10 deaths documented, corresponding to a mortality rate of 0.680%. This improvement in the epidemiological situation coincided with the rollout of Chad's vaccination program, which began on June 4, 2021, with an initial allocation of 200 000 doses of the Sinopharm BIBP vaccine donated by China. It is plausible that the vaccination efforts, alongside other public health measures, contributed to the observed reduction in morbidity and mortality rates. However, further data are needed to fully assess the extent of their impact. Additionally, this improvement occurred despite an increase in the urban population, which grew to approximately 1.533 million inhabitants by the end of 2022. In this study, we integrate seroprevalence data and whole genome sequencing to provide a preliminary understanding of the COVID‐19 epidemic, particularly in N'Djamena, identified as Chad's primary COVID‐19 hotspot.

## 2 Materials and Methods

### 2.1 Data and Sample Collection

From August 12 to October 26, 2021, immediately following Chad's second epidemic wave as documented by the World Health Organization [8], we conducted an investigation involving participants from 11 selected hospitals and public health facilities in N'Djamena. During this period, we collected 2700 samples from voluntary donors who attended these healthcare facilities for routine consultations and subsequently participated in laboratory blood sampling. To expand our 2022 data set, we incorporated an additional 176 samples from voluntary donors at King Faycal Hospital—a facility distinguished by its considerable contribution during the 2021 data collection and its strategic location proximate to the Grand Mosque and Central Market. These supplementary samples functioned as controls for the 2022 N'Djamena data set. Data collection was facilitated through an electronic questionnaire on KoboToolBox, targeting potential risk factors associated with participants. Whole blood was collected in BD VACUTAINER K3 EDTA tubes, and plasma was separated by centrifugation at 3000 rpm for 10 min at room temperature. Plasma aliquots were stored in cryotubes at −80°C until use, while hemolyzed blood was excluded. Laboratory analyses were carried out in N'Djamena at both the Laboratory for Major Tropical Epidemics (LAGET) and the Institute for Research on Livestock and Development (IRED)in N'Djamena.

### 2.2 Detection of Anti‐SARS‐CoV‐2 Nucleocapsid Protein

The detection of plasma IgG antibodies against the SARS‐CoV‐2 Nucleocapsid (N) antigen was carried out on each sample using the indirect ELISA (enzyme‐linked immunosorbent assay) method, according to manufacturer's instructions (DIATHEVA kit, www.diatheva.com). In brief, the sample's anti‐N SARS‐CoV‐2 IgG antibodies bind specifically to the N SARS‐CoV‐2 antigen. and to the horseradish peroxidase (HRP) conjugated secondary antibodies. This immune complex was visualized by incorporating the substrate ABTS (2,2'‐Azino‐bis 3‐ethylbenzothiazoline‐6‐sulfonic acid), resulting in a green reaction product detected by absorbance measure at 405 nm. Results were determined based on the ratio between the cut‐off value (Co) and sample absorbance (S), with a sample being deemed positive when the S/Co ratio was ≥ 1.5. According to the manufacturer, the test's sensitivity stood at 98.80%, and its specificity was 98.02%.

### 2.3 Detection of Total Anti‐RBD Antibodies for SARS‐CoV‐2 (Roche Method)

In 10% of the samples (*n* = 288), the presence of total SARS‐CoV‐2 antibodies targeting the spike protein was assessed using the Electro‐chemiluminescence immunoassay double‐antigen sandwich method via the Elecsys Anti‐SARS‐CoV‐2 S assay. These tests were conducted in line with the manufacturer's guidelines provided by Elecsys Anti‐SARS‐CoV‐2 S (Roche Diagnostics, GmbH) and utilized the Cobas e 411 immunoassay analyzer. Results were automatically reported as the analyte concentration of each sample in U/mL, with < 0.80 U/mL interpreted as negative for anti‐SARS‐CoV‐2 S antibodies and ≥ 0.80 U/mL interpreted as positive for anti‐SARS‐CoV‐2 S antibodies.

### 2.4 Anti‐Spike Antibody Detection Using the VSV Pseudo‐Neutralization Assay

For a pooled sample of 100 plasma specimens, created by pooling together 10 individual samples, an in vitro neutralization assay was conducted to evaluate the capability of antibodies to neutralize the replication of a vesicular stomatitis pseudovirus (VSV‐pseudovirus) bearing the SARS‐CoV‐2 spike protein (Spike‐VSV) in VeroE6 cells. The pseudotype virus used was a single‐cycle, replication‐incompetent VSV pseudovirus. Neutralization was detected based on GFP expression, which indicates the level of viral inhibition by the antibodies. The VeroE6 epithelial cell line, originating from the kidney of the African green monkey, Cercopithecus aethiops, was maintained in Dulbecco's modified Eagle's medium enriched with 10% fetal bovine serum, an antibiotic‐antimycotic combination, Hepes buffer, L‐glutamine, and gentamicin. Cultures were maintained at 37°C under a 5% CO_2_ humidified atmosphere. The virus titer was determined using the Reed and Muench method [11] and reported as the 50% tissue culture infective dose (TCID50/mL). Antibody dilutions tested ranged from 1:20 to 1:2560. Samples were pooled to ensure sufficient volume and to evaluate the average neutralizing antibody response within the samples analyzed.

### 2.5 Seroprevalence Data Analysis

Data analysis was conducted using SPSS version 20. Pearson's chi‐squared test was employed to assess associations between variables, with statistical significance determined at a 95% confidence interval. The *p*‐value for statistical significance was set at < 0.05. In all relevant tables, the *p*‐values and confidence intervals are clearly indicated and calculated using standard methods appropriate for chi‐squared tests.

### 2.6 Whole‐Genome Sequencing and Bioinformatic Analysis of Selected SARS‐CoV‐2 Positive Samples

In the period between Chad's second and third SARS‐CoV‐2 epidemic waves (July 2021–January 2022), we collected nasopharyngeal swab samples (2–3 mL) from patients confirmed positive for COVID‐19 through quantitative reverse transcriptase‐polymerase chain reaction (qRT‐PCR). For this study, we selected samples with a threshold cycle of less than 30 (*Ct* < 30). A total of 42 samples, collected between 2021 and 2022 (*n* = 22 from 2021 and *n* = 20 from 2022), were obtained from five molecular diagnostic facilities in N'Djamena: (i) Laboratoire de Biosécurité et des Epidémies, (ii) Laboratoire de Centre Hospitalier la Référence, (iii) Laboratoire de Grandes Epidemies Tropicales (LAGET) de Complexe Hospitalo—Universitaire Bon Samaritain, (iv) Laboratoire National de la TB, and (v) Laboratoire de l'Hôpital Militaire d'Instruction. The clinical severity of the patients was assessed following the World Health Organization (WHO) guidelines outlined in the Clinical Management of COVID‐19 Patients—Interim Guidance. Cases were categorized into four groups: mild, moderate, severe, and critical. Among the 42 cases, the majority (35) were categorized as mild. Seven cases were classified as severe, presenting with respiratory distress or difficulty. Notably, none of the cases were classified as critical, and no patients required intensive care.

### 2.7 RNA Extraction and Molecular Diagnostic Assay

Viral RNA was extracted from inactivated oro and nasopharyngeal swabs using the QIAmp Viral RNA Mini Kit (Qiagen) and tested for SARS‐CoV‐2 by multiplex real‐time PCR assays using the Allplex 2019‐nCoV Assay (Seegene) targeting the envelope (E), the RNA dependent RNA polymerase (RdRp) and the nucleocapsid (N) genes.

### 2.8 cDNA Synthesis and Whole Genome Sequencing

Samples were selected for sequencing based on the *Ct* value (≤ 30) and availability of epidemiological metadata, such as date of sample collection, sex, age, and municipality of residence. Whole genome sequencing was carried out using the Oxford Nanopore Technology [12]. Following the manufacturer's instructions, the SuperScript IV Reverse Transcriptase reagent (Invitrogen) was initially used for cDNA synthesis. Sequencing multiplex PCR was performed on the cDNA using the Q5 High Fidelity Hot‐Start DNA Polymerase (New England Biolabs) and a set of specific primers designed by the ARTIC Network for sequencing the complete SARS‐CoV‐2 genome (version 4.1) [12]. All experiments were conducted in a level‐2 biosafety cabinet. Amplicons were purified with 1x AMPure XP Beads (Beckman Coulter) and quantified with QubitTM dsDNA HS Assay Kit (Thermofisher Scientific) on a Qubit 3.0 fluorimeter (Thermofisher Scientific). DNA library preparation was performed using the Ligation Sequencing Kit LSK109 (Oxford Nanopore Technologies) and the Native Barcoding Kit (NBD104 and NBD114, Oxford Nanopore Technologies). Sequencing libraries were loaded into an Oxford Nanopore Technologies R9.4 flow cell. In each sequencing run, we employed negative controls to prevent and detect potential contamination with mean coverage of less than 2%. Guppy v3.4.5 was used to basecall sequencing raw files, and qcat was used to demultiplex barcodes. Using Genome Detective, consensus sequences were generated by de novo assembly [13].

### 2.9 Phylogenetic Analysis

Lineage assignment was carried out using the PANGOLIN tool v.4.3 (Phylogenetic Assignment of Named Global Outbreak Lineages) [14]. The new sequences generated in this study (*n* = 42) were compared with a diverse set of genomic sequences (*n* = 3000). Given the large amount of genome sequences available on public repositories, we used the Subsampler tool (available at https://github.com/andersonbrito/subsampler) which is in turn a pipeline for sub‐sampling genomic data based on epidemiological series. Sequences were aligned with ViralMSA using the default parameters [15]. The alignment was manually cleaned to remove artifacts in terminal regions using Aliview [16]. The phylogenetic analysis was carried out using the maximum likelihood method implemented in the IQ‐TREE v.2 software [17]. The model tree was deduced using the general model method of time‐reversible nucleotide substitution (GTR) and a proportion of invariant sites (+I) selected by the ModelFinder application. Branch support was assessed by the bootstrap‐based approximate likelihood ratio test and Shimon's test. The ML tree was then transformed into time‐scale phylogenies in TreeTime using a standard mutation rate of 0.0008 substitutions per site per year and a standard clock deviation of 0.0004 [12, 18].

### 2.10 Epidemiology Genomic Data Assembly

We analyzed COVID‐19 genomes in Chad from publicly released data up to March 1, 2023, collected from GISAID (https://gisaid.org/) and used RStudio for data visualization.

### 2.11 Ethical Consideration

The study received ethical approval by “Comité National de Bioéthique du Tchad (CNBT)” n° 011CMT/PC/PMT/MESRI/SG/CNBT/2022. All volunteer participants were provided with an informational document, and informed consent was diligently obtained, extending to minors as well. For participants below the legal age of consent, informed consent was obtained from their respective parents or legal guardians. Additionally, to ensure the confidentiality of the collected data, each participant was assigned a unique code. Additionally, all techniques were followed in compliance with the applicable norms and legislation.

## 3 Results

### 3.1 Seroprevalence of COVID‐19 in Chad

Our first comprehensive seroprevalence assessment of SARS‐CoV‐2 was executed in 2021 across 11 healthcare facilities within the city of N'Djamena, using the ELISA technique. Of the 2700 samples tested, an overall seroprevalence rate of 69.5% (95% confidence interval [CI]: 67.7–71.3) was observed, identifying 1877 seropositive individuals. A subset analysis of 10% of the samples, focused on the presence of total SARS‐CoV‐2 anti‐RBD antibodies targeting the spike protein, revealed a positivity rate of 94.8% (*n* = 239/252) (Table 1). Furthermore, a select batch of plasma samples, when subjected to neutralization tests against the SARS‐CoV‐2 spike protein, indicated that 59.0% (*n* = 59/100) tested positive for neutralizing antibodies. Transitioning to 2022, the seroprevalence assessment underscored the continued and intensive circulation of the SARS‐CoV‐2 virus among N'Djamena's asymptomatic residents. The seroprevalence rate increased from 68.8% in 2021 to 85.8% in 2022, representing an approximate growth of 17% (Table 1).

**Table 1:** Seroprevalence of COVID‐19 in N'Djamena Chad.

Antibodies tested (year)	Positive	*N*	Seroprevalence %	95% CI lower	95% CI
Overall anti‐N (2021)	1877	2700	69.5	67.7	71.3
IgG anti‐RBD IgG (2021)	239	252	94.8	91.3	96.9
Neutralizing antibodies (2021)	59	100	59	49.2	68.1
Anti‐N (2021)	262	381	68.8	63.9	73.2
Anti‐N (2022)	151	176	85.8	79.9	90.2

*Note: N*: total number tested, %: percentage, CI: confidence interval, *p* < 0.05 is significant, anti‐N: IgG antibodies against SARS‐CoV‐2 nucleocapsid, anti total RBD: IgG, IgM, IgA antibodies against SARS‐CoV‐2 receptor binding domain.

## 4 Genomic Evaluation and Phylogenetic Inference

**Figure 1:**
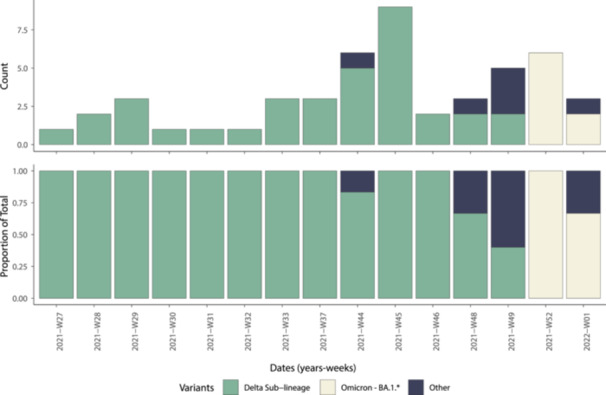
Genetic diversity and sequencing statistics of SARS‐CoV‐2. The chart illustrates the percentage of the SARS‐CoV‐2 genome sequenced in relation to the RT‐qPCR *Ct* value for each of the 42 samples. Each circle represents a sequence recovered from an infected individual in Chad, with colors indicating lineage assignment.

To investigate the transmission dynamics of SARS‐CoV‐2 variants circulating in the city of N'Djamena, we sequenced 42 near‐full genomes from SARS‐CoV‐2 RT‐qPCR positive samples collected between November 2021 and January 2022. The participants included 16 females and 26 males, with a median age of 38.0 years (range: 6–69 years) (Table 2). All samples had sufficient viral genetic material (≥ 2 ng/µL) for library preparation. The average PCR cycle threshold (*Ct*) value for these samples was 19.50 (range: 13–28). The sequenced genomes had a median coverage of 92% (range: 79–99). A notable relationship was observed between *Ct* values and genome coverage: samples with lower *Ct* values, which indicate a higher viral load, typically had better average genome coverage (Figure 1).

**Table 2:** Genomic and clinical characteristics of COVID‐19 cases in Chad, stratified by lineage and symptom severity.

IDs	Country	Region	Collection‐date	CT‐value	Coverage	Sex	Age	GISAID‐ID	Lineage	Symptoms
Chad|20220316_49744/ARTIC/medaka|2021‐11‐23	Chad	N'Djamena	2021‐11‐23	18.76	0.9798	F	46	EPI_ISL_18731672	Delta	Mild
Chad|20220316_49694/ARTIC/medaka|2021‐11‐23	Chad	N'Djamena	2021‐11‐23	19.59	0.9086	M	58	EPI_ISL_18731673	Delta	Mild
Chad|20220316_49673/ARTIC/medaka|2021‐11‐23	Chad	N'Djamena	2021‐11‐23	16.78	0.993	F	6	EPI_ISL_18731674	Delta	Mild
Chad|20220316_49670/ARTIC/medaka|2021‐11‐23	Chad	N'Djamena	2021‐11‐23	17.41	0.964	F	29	EPI_ISL_18731675	Delta	Mild
Chad|20220316_49659/ARTIC/medaka|2021‐11‐23	Chad	N'Djamena	2021‐11‐23	18.26	0.8464	M	39	EPI_ISL_18731676	B.1.640	Mild
Chad|20220316_49645/ARTIC/medaka|2021‐11‐23	Chad	N'Djamena	2021‐11‐23	15.26	0.9425	M	57	EPI_ISL_18731677	Delta	Mild
Chad|20220316_49431/ARTIC/medaka|2021‐11‐19	Chad	N'Djamena	2021‐11‐19	20.28	0.9385	M	40	EPI_ISL_18731678	Delta	Mild
Chad|20220316_48913/ARTIC/medaka|2021‐11‐15	Chad	N'Djamena	2021‐11‐15	14.83	0.9827	M	33	EPI_ISL_18731679	Delta	Mild
Chad|20220316_48864/ARTIC/medaka|2021‐11‐15	Chad	N'Djamena	2021‐11‐15	19.75	0.9741	M	60	EPI_ISL_18731680	Delta	Mild
Chad|20220316_48511/ARTIC/medaka|2021‐11‐12	Chad	N'Djamena	2021‐11‐12	18.21	0.9877	F	32	EPI_ISL_18731681	Delta	Mild
Chad|20220316_48458/ARTIC/medaka|2021‐11‐11	Chad	N'Djamena	2021‐11‐11	19.1	0.9518	M	43	EPI_ISL_18731682	Delta	Mild
Chad|20220316_48400/ARTIC/medaka|2021‐11‐11	Chad	N'Djamena	2021‐11‐11	17.29	0.9663	F	50	EPI_ISL_18731683	Delta	Mild
Chad|20220316_48139/ARTIC/medaka|2021‐11‐11	Chad	N'Djamena	2021‐11‐11	13.56	0.9935	F	33	EPI_ISL_18731684	Delta	Mild
Chad|20220316_48035/ARTIC/medaka|2021‐11‐10	Chad	N'Djamena	2021‐11‐10	16.5	0.9937	M	15	EPI_ISL_18731685	Delta	Mild
Chad|20220316_48032/ARTIC/medaka|2021‐11‐10	Chad	N'Djamena	2021‐11‐10	14.17	0.9725	F	32	EPI_ISL_18731686	Delta	Severe
Chad|20220316_47910/ARTIC/medaka|2021‐11‐09	Chad	N'Djamena	2021‐11‐09	24.29	0.9027	F	45	EPI_ISL_18731687	Delta	Severe
Chad|20220316_47815/ARTIC/medaka|2021‐11‐09	Chad	N'Djamena	2021‐11‐09	23.22	0.7959	F	54	EPI_ISL_18731688	Delta	Mild
Chad|20220303_53639/ARTIC/medaka|2022‐01‐07	Chad	N'Djamena	2022‐01‐07	28.24	0.897	f	43	EPI_ISL_18731689	Omicron BA.1	Mild
Chad|20220303_53543/ARTIC/medaka|2022‐01‐05	Chad	N'Djamena	2022‐01‐05	18.25	0.9353	M	66	EPI_ISL_18731690	Omicron BA.1	Severe
Chad|20220303_53366/ARTIC/medaka|2022‐01‐05	Chad	N'Djamena	2022‐01‐05	16.24	0.9334	M	13	EPI_ISL_18731691	Omicron BA.1	Severe
Chad|20220303_51504/ARTIC/medaka|2021‐12‐02	Chad	N'Djamena	2021‐12‐02	21.24	0.8429	F	38	EPI_ISL_18731692	Delta	Mild
Chad|20220303_51439/ARTIC/medaka|2021‐12‐02	Chad	N'Djamena	2021‐12‐02	20.24	0.8232	M	47	EPI_ISL_18731693	B.1.640	Mild
Chad|20220303_51046/ARTIC/medaka|2021‐12‐02	Chad	N'Djamena	2021‐12‐02	16.24	0.952	M	27	EPI_ISL_18731694	Delta	Mild
Chad|20220303_50973/ARTIC/medaka|2021‐12‐02	Chad	N'Djamena	2021‐12‐02	17.22	0.8038	F	53	EPI_ISL_18731695	B.1.640	Mild
Chad|20220303_50926/ARTIC/medaka|2021‐12‐02	Chad	N'Djamena	2021‐12‐02	20.24	0.8086	M	43	EPI_ISL_18731696	Delta	Mild
Chad|20220301_54296/ARTIC/medaka|2022‐01‐24	Chad	N'Djamena	2022‐01‐24	20.96	0.9276	m	42	EPI_ISL_18731697	Omicron BA.1	Severe
Chad|20220301_54281/ARTIC/medaka|2022‐01‐24	Chad	N'Djamena	2022‐01‐24	24.78	0.931	m	29	EPI_ISL_18731698	Omicron BA.1	Mild
Chad|20220301_54223/ARTIC/medaka|2022‐01‐18	Chad	N'Djamena	2022‐01‐18	21.68	0.9261	M	29	EPI_ISL_18731699	Omicron BA.1	Severe
Chad|20220301_54222/ARTIC/medaka|2022‐01‐18	Chad	N'Djamena	2022‐01‐18	25.14	0.907	m	30	EPI_ISL_18731700	Omicron BA.1	Severe
Chad|20220301_54170/ARTIC/medaka|2022‐01‐13	Chad	N'Djamena	2022‐01‐13	13.07	0.9274	F	49	EPI_ISL_18731701	Omicron BA.1	Mild
Chad|20220301_54168/ARTIC/medaka|2022‐01‐13	Chad	N'Djamena	2022‐01‐13	18.09	0.9284	F	28	EPI_ISL_18731702	Omicron BA.1	Mild
Chad|20220301_53913/ARTIC/medaka|2022‐01‐13	Chad	N'Djamena	2022‐01‐13	20.93	0.9302	M	28	EPI_ISL_18731703	Omicron BA.1	Mild
Chad|20220301_53908/ARTIC/medaka|2022‐01‐12	Chad	N'Djamena	2022‐01‐12	13.9	0.9213	M	31	EPI_ISL_18731704	Omicron BA.1	Mild
Chad|20220301_53905/ARTIC/medaka|2022‐01‐13	Chad	N'Djamena	2022‐01‐13	16.67	0.9174	M	58	EPI_ISL_18731705	Omicron BA.1	Mild
Chad|20220301_53891/ARTIC/medaka|2022‐01‐11	Chad	N'Djamena	2022‐01‐11	24.6	0.9262	M	17	EPI_ISL_18731706	Omicron BA.1	Mild
Chad|20220301_53885/ARTIC/medaka|2022‐01‐13	Chad	N'Djamena	2022‐01‐13	21.3	0.9353	M	31	EPI_ISL_18731707	Omicron BA.1	Mild
Chad|20220301_53876/ARTIC/medaka|2022‐01‐13	Chad	N'Djamena	2022‐01‐13	19.51	0.9353	F	35	EPI_ISL_18731708	Omicron BA.1	Mild
Chad|20220301_53827/ARTIC/medaka|2022‐01‐11	Chad	N'Djamena	2022‐01‐11	17.4	0.966	M	69	EPI_ISL_18731709	Omicron BA.1	Mild
Chad|20220301_53812/ARTIC/medaka|2022‐01‐11	Chad	N'Djamena	2022‐01‐11	27.63	0.9266	M	36	EPI_ISL_18731710	Omicron BA.1	Mild
Chad|20220301_53811/ARTIC/medaka|2022‐01‐11	Chad	N'Djamena	2022‐01‐11	26.63	0.8677	M	55	EPI_ISL_18731711	Omicron BA.1	Mild
Chad|20220301_53807/ARTIC/medaka|2022‐01‐11	Chad	N'Djamena	2022‐01‐11	21.63	0.9068	F	43	EPI_ISL_18731712	Omicron BA.1	Mild
Chad|20220301_53786/ARTIC/medaka|2022‐01‐10	Chad	N'Djamena	2022‐01‐10	19.63	0.8694	M	7	EPI_ISL_18731723	Omicron BA.1	Mild

**Figure 2:**
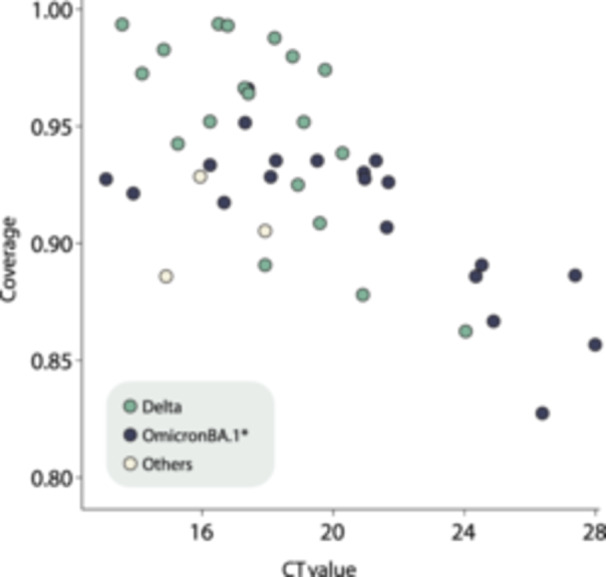
Temporal distribution of SARS‐CoV‐2 variants in N'Djamena, Chad from November 2021 to January 2022.

**Figure 3:**
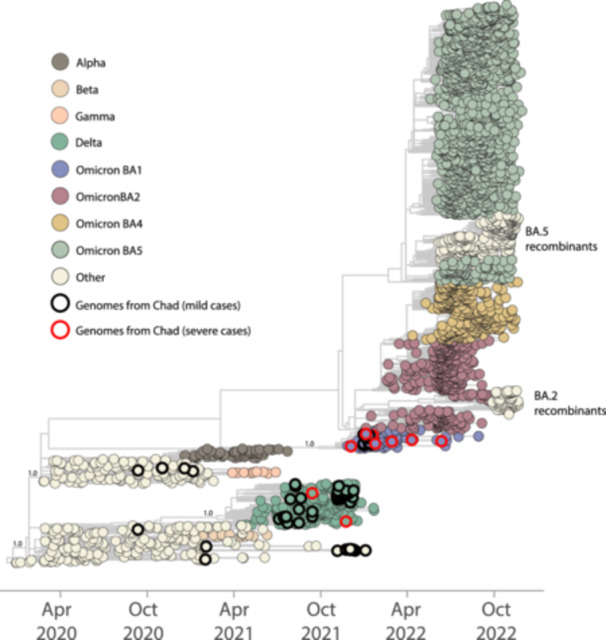
Phylogenetic analysis of SARS‐CoV‐2 viral strains circulating in N'Djamena, Chad, between November 2021 and January 2022. A time‐resolved maximum‐likelihood tree of SARS‐CoV‐2, including novel isolates (*n* = 42) and representative worldwide subsample genomes (*n* = 3000) collected up to November 2022. The genomes are colored according to the lineages (VOC and ancestral lineages) as shown in the legend on the top left. The genomes generated in this study have been highlighted in the tree with circles: black circles represent genomes from patients with mild cases, while red circles represent genomes from patients with severe cases.

Table 2 provides detailed epidemiological data and sequencing statistics for the generated sequences. Using the dynamic nomenclature established for SARS‐CoV‐2 lineages, we identified the sequences as belonging to three distinct SARS‐CoV‐2 strains: (i) B.1.640, originally identified in the Republic of Congo and, in this study, found in a limited number of strains (*n* = 3, as detailed in Table 2); (ii) Delta; and (iii) Omicron BA.1* PANGO‐lineages. Both Delta and Omicron BA.1* lineages, widely recognized on a global scale, have significantly influenced the transmission dynamics of SARS‐CoV‐2 in Chad, as visualized in Figures 2 and 3. Despite the small number of whole‐genome sequences in our investigation, our findings are consistent with previous research. They emphasize the pandemic's viral evolution, in which Omicron‐related lineages (BA.1*) rapidly displaced the previously dominant Delta variation, as seen in Figure 2. The replacement of dominant lineages during the pandemic reflected the effectiveness of SARS‐CoV‐2 intrinsic mechanisms of adaptation facing the host immune system, whether triggered by vaccination or not, as well as the effects of previous levels of infection by other VOCs across populations. Phylogenetic analysis, combining our novel isolates with a representative data set from GISAID (https://www.gisaid.org/) up to November 2022, demonstrated that the country experienced multiple introduction events. This underscores how human mobility significantly influenced the progression of the SARS‐CoV‐2 epidemic in the country (Figure 3). These introductions often resulted in local transmissions, especially during periods of high infection rates. However, the limited availability of complete SARS‐CoV‐2 genome sequences from Chad has a significant impact on our estimates and ability to fully characterize the molecular epidemiology of these variants at a regional level. This highlights the need to increase sequencing efforts to reduce disparities and improve real‐time data generation, sharing, and representativeness, as emphasized by previous studies.

## 5 Discussion

The COVID‐19 pandemic has underscored the importance of regionalized understandings of transmission dynamics, as variations in disease spread and outcomes across different areas can inform localized public health interventions [19]. Our findings from N'Djamena, Chad's capital, shed light on the epidemiological intricacies of one such region that became a significant hotspot for COVID‐19 [20]. One of the most striking results from our research is the disparity between confirmed COVID‐19 case counts and the seroprevalence data. Preliminary indications from the seroprevalence data highlight a vast portion of the N'Djamena population that might have been exposed to the virus. Such a discrepancy might be attributed to underreporting of cases, limited testing capacities, or a significant number of asymptomatic infections [21, 22, 23, 24]. On this respect, one likely potential link might be the previous exposures to viruses similar to SARS‐CoV‐2, such as MERS (Middle East Respiratory Syndrome) [25]. Such prior exposures could have played a role in modulating the immune response to SARS‐CoV‐2, leading to milder disease manifestations and thereby contributing to the observed discrepancy in case counts and seroprevalence data. Furthermore, the concept of herd immunity, not just for COVID‐19 but also from other infectious agents, cannot be overlooked. The population might exhibit some level of protection from prior infections, either by similar coronaviruses or other pathogens, which can influence the transmission dynamics of newer pathogens like SARS‐CoV‐2 [26]. Lastly, it's worth noting the potential impact of traditional medicinal practices on disease outcomes. There have been recent discussions in the region about the use of medicinal plant infusions with antiviral and inflammatory properties, as proposed by Mutombo et al. [27] and Afro [28]. These natural remedies might have contributed to alleviating some of the symptoms or even boosting immunity against the virus, which requires further investigation. In our study, we employed a combined approach of seroprevalence data collection and whole genome sequencing of SARS‐CoV‐2 to comprehensively assess both the extent of population exposure and the genetic diversity of circulating virus strains. Relevantly, the genome sequencing revealed the co‐circulation of multiple SARS‐CoV‐2 strains in N'Djamena. With some of these strains being identified as global variants of concern, it's imperative to delve deeper into their implications for transmission efficiency, disease severity, and potential resistance to existing vaccines [29]. The dynamic interplay among these co‐circulating strains could have significantly shaped the epidemic's trajectory in the region. Furthermore, a crucial aspect that needs consideration is human mobility and the connectiveness of regions. The movement of people across borders, whether for trade, tourism, or other purposes, can greatly facilitate the introduction and spread of these viral strains into previously unaffected or less‐affected regions. In cities like N'Djamena, where economic and social activities often transcend national borders, the role of human mobility becomes even more pronounced [30]. Continuous monitoring and understanding of migration patterns can offer insights to public health authorities to prevent potential outbreaks and implement effective containment strategies [31]. The integrated, multidisciplinary approach we've employed in this study underlines the significance of combining diverse epidemiological tools. By investigating the epidemic from both a serological and genomic standpoint, we have a more comprehensive understanding of the disease's spread and its potential implications. In conclusion, our research emphasizes the indispensable role of comprehensive epidemiological research in formulating precise public health interventions. As the world faces the challenges posed by infectious diseases, particularly those of pandemic potential such as COVID‐19, a deeper regional understanding can facilitate the development of more effective, targeted strategies, ensuring the health of communities.

## Limitations of the Study

One of the primary vulnerabilities in our manuscript comes around the challenges associated with the sequencing process. Due to preservation difficulties, there was a substantial limitation in the sequence coverage, leading to a reduction in the number of samples sequenced. Furthermore, the inconsistencies and gaps identified in the documentation of clinical data underscore the necessity for a more rigorous data collection approach. These combined factors may have impacted the depth of our insights, and future endeavors would benefit from addressing these challenges to ensure a more holistic and accurate representation of the local epidemiological situation.

## Author Contributions

**Mathiew Hota:** conceptualization, methodology, investigation, original draft preparation, review and editing, supervision. **Andrillene L. D. Wondeu:** methodology, investigation, review and editing. **Mahamat F. Abakar:** methodology, investigation, review and editing. **Koutaya Dezoumbe:** methodology, investigation, review and editing. **Fatima Abdelrazakh:** methodology, investigation, review and editing. **Sabrina Atturo:** methodology, investigation, review and editing. **Nathan Naïbeï:** methodology, investigation, review and editing. **Giulia Cappelli:** methodology, investigation, review and editing. **Franck Mennechet:** methodology, investigation, review and editing. **Fissou H. Yandai:** methodology, investigation, review and editing. **Djamal H. Abdallah:** methodology, investigation, review and editing. **Zongo R. F. Edgard:** methodology, investigation, review and editing. **Abdoulaye Boukar:** methodology, investigation, review and editing. **Choroma A. Moussa:** methodology, investigation, review and editing. **Issa M. Yaya:** methodology, investigation, review and editing. **Mahamat I. Hamad:** methodology, investigation, review and editing. **Nontegyol Armand:** methodology, investigation, review and editing. **Netalar Honorine:** methodology, investigation, review and editing. **Kayanlengar Frederic:** methodology, investigation, review and editing. **Adam A. Moustapha:** methodology, investigation, review and editing. **Yanda M. Daniel:** methodology, investigation, review and editing. **Adam M. Alim:** methodology, investigation, review and editing. **Mahamat Grene:** methodology, investigation, review and editing. **Oumaima Djarma:** methodology, investigation, review and editing. **Noubaramadji Y. Suitombaye:** methodology, investigation, review and editing. **Amine Akouya:** methodology, investigation, review and editing. **Ouchemi Choua:** methodology, investigation, review and editing. **Guy R. T. Dzomo:** methodology, investigation, review and editing. **Djallaye Djimtoïbaye:** methodology, investigation, review and editing. **Vittorio Colizzi:** conceptualization, methodology, investigation, original draft preparation, review and editing, supervision. **Mahamat A. Moussa:** conceptualization, methodology, investigation, original draft preparation, review and editing, supervision. **Marta Giovanetti:** conceptualization, methodology, software, investigation, data curation, original draft preparation, review and editing, visualization, supervision. All authors have read and agreed to the published version of the manuscript.

## Conflicts of Interest

The authors declare no conflicts of interest.

## Data Availability

The newly generated sequences have been submitted to the GISAID Public Repository, accessible at [https://www.gisaid.org/]. The IDs for these sequences (EPI_ISL_18731672 to EPI_ISL_18731723) are also listed in Table 2, along with all associated metadata.
